# Green Tea Polyphenol Epigallocatechin-3-Gallate Enhance Glycogen Synthesis and Inhibit Lipogenesis in Hepatocytes

**DOI:** 10.1155/2013/920128

**Published:** 2013-08-27

**Authors:** Jane J. Y. Kim, Yi Tan, Linda Xiao, Ya-Li Sun, Xianqin Qu

**Affiliations:** ^1^School of Medical and Molecular Biosciences, University of Technology, Sydney, P.O. Box 123, NSW 2007, Australia; ^2^Medical Association of Minorities, State Administration of Traditional Medicine, China

## Abstract

The beneficial effects of green tea polyphenols (GTP) against metabolic syndrome and type 2 diabetes by suppressing appetite and nutrient absorption have been well reported. However the direct effects and mechanisms of GTP on glucose and lipid metabolism remain to be elucidated. Since the liver is an important organ involved in glucose and lipid metabolism, we examined the effects and mechanisms of GTP on glycogen synthesis and lipogenesis in HepG2 cells. Concentrations of GTP containing 68% naturally occurring (−)-epigallocatechin-3-gallate (EGCG) were incubated in HepG2 cells with high glucose (30 mM) under 100 nM of insulin stimulation for 24 h. GTP enhanced glycogen synthesis in a dose-dependent manner. 10 **μ**M of EGCG significantly increased glycogen synthesis by 2fold (*P* < 0.05) compared with insulin alone. Western blotting revealed that phosphorylation of Ser9 glycogen synthase kinase 3**β** and Ser641 glycogen synthase was significantly increased in GTP-treated HepG2 cells compared with nontreated cells. 10 **μ**M of EGCG also significantly inhibited lipogenesis (*P* < 0.01). We further demonstrated that this mechanism involves enhanced expression of phosphorylated AMP-activated protein kinase **α** and acetyl-CoA carboxylase in HepG2 cells. Our results showed that GTP is capable of enhancing insulin-mediated glucose and lipid metabolism by regulating enzymes involved in glycogen synthesis and lipogenesis.

## 1. Introduction

Metabolic syndrome is a complex cluster of several metabolic abnormalities that significantly increases the risk of cardiovascular disease and the onset of type 2 diabetes (T2D). Metabolic syndrome has become a significant public health problem, affecting millions of people all over the world [1]. Treatment of metabolic syndrome is crucial to public health. It involves improving underlying insulin resistance and central obesity in individuals through oral medications and lifestyle modifications [2] such as increasing physical activity and promoting healthy diets. If left unresolved in an individual, metabolic syndrome may progress to T2D and cardiovascular morbidity.

After water, tea is the most widely consumed beverage in the world. Consumption of green tea (*Camellia sinensis*, Theaceae) in particular is reported to have various beneficial effects on metabolic syndrome. One such example of the beneficial effects of green tea consumption is its role in promoting fat oxidation, which is a key preventative of obesity in healthy individuals [3]. Another example of the benefits of green tea consumption is its observed effect in reducing the occurrence of T2D in both laboratory animal studies [4, 5] and clinical studies involving subjects with metabolic syndrome or prediabetes [6, 7] by improving insulin sensitivity and glucose tolerance.

Several lines of studies suggest that the antidiabetic effects of green tea consumption are probably due to the effect of green tea (or more specifically, its active ingredients) in lowering central obesity—a major component of metabolic syndrome—by suppressing appetite and nutrient absorption [8, 9]. Yang et al. and Muramatsu et al. [10, 11] report that polyphenolic compounds found in green tea extract such as (−)-epigallocatechin-3-gallate (EGCG) increase fecal lipid content in high fat-fed rats. EGCG has also been shown to increase fecal cholesterol excretion and fecal fat excretion in high fat, high cholesterol-fed rats when compared with the control group [9]. Considerable amount of data demonstrate that one mechanism by which tea polyphenols act against obesity and hyperlipidemia is by modifying dietary fat emulsification in the gastrointestinal tract and inhibiting of gastrointestinal lipolysis [12, 13]. However, it has been recently observed that polyphenols in green tea are also capable of accessing the bloodstream through the intestinal epithelial outer cell membrane [14]. Due to a lack of study on this observation, questions remain as to the effect of any direct actions and mechanisms of green tea consumption on glucose and lipid metabolisms in insulin-targeted tissues and organs. 

The liver is a major organ involved in glucose and lipid metabolism. Insulin resistance in the liver leads to increased hepatic glucose production and lipogenesis, which contributes to hyperglycemia and lipotoxicity-induced pancreatic *β*-cell dysfunction [15]. To understand the direct effects and mechanisms of green tea polyphenols (GTP) on regulating glucose and lipid metabolism in the liver, we examined the dose-response effect of GTP on glycogen synthesis and lipogenesis in human hepatoma HepG2 cells. HepG2 cells are considered suitable cellular models for examining glycogen synthesis and lipogenesis in the liver [16]. 

## 2. Materials and Methods

### 2.1. Green Tea Polyphenols and (−)-Epigallocatechin-3-Gallate

**Figure 1:**
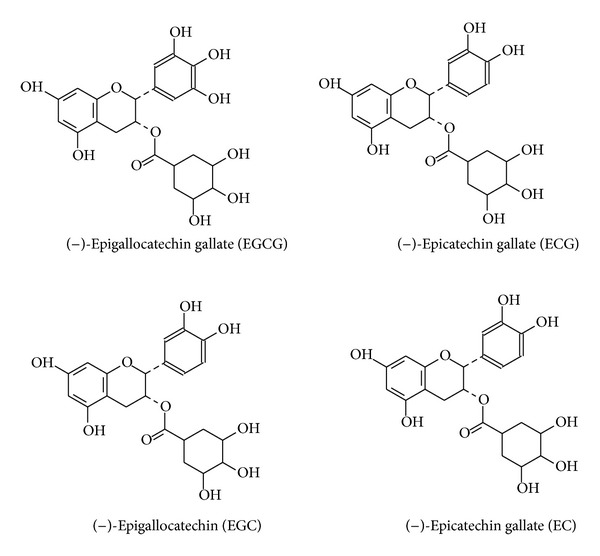
Structures for four polyphenols in green tea.

The tea polyphenols (99% purity) were extracted from green tea leaves grown in Guizhou province, South-Western China, by Zuyi Lushen Kangyuan Co (Guizhou, Meitan, China). The polyphenolic compounds identified using liquid chromatography-mass spectrometry (LC-MS) were 68% EGCG, 7% epigallocatechin (EGC), 1% epicatechin gallate (ECG), and 19% epicatechin (EC) in GTP (w/w), the structures of these polyphenols are shown in Figure 1. According to the literature, the most potent bioactive catechin in GTP is EGCG, followed by ECG, and EGC and EC with weak biological action [17]. To observe dose-response relationship of GTP on glycogen synthesis and lipogenesis, the molecular weight of EGCG was used to calculate a series of mole concentrations (0.01, 0.1, 1, and 10 *μ*M) to present the total GTP (1 : 0.68 GTP versus EGCG) for the cellular study mentioned later and the term of GTP-EGCG was used to present all results from total polyphenols used in this study.

### 2.2. Cell Culture and Treatment

Human hepatoma HepG2 cells (ATCC HB 8065, ATCC, VA, USA) were maintained in Dulbecco's modified eagle medium (DMEM) containing normal glucose (5 mM glucose), supplemented with 10% fetal bovine serum (FBS), and 100 U/mL penicillin (GIBCO, Aukland, NZ) in an incubator (37°C and 5% CO_2_). HepG2 cells were grown in complete media (CM) with 10% FBS until 70% cell confluence was reached. 24 h prior to all experimental procedures, appropriate glucose concentrations (5 mM or 30 mM D-glucose) were added to cells. 

Approximately 4 × 10^3^ cells were seeded to 24-well plates for all assays. When confluent, CM was discarded and then starving medium (SM) containing 0.5% FBS was added. After 6 h incubation with SM, 100 nM of insulin (Eli Lilly Pty Ltd, NSW, Australia) was added in each well, followed by adding a range of 0.01–10 *μ*M EGCG into appropriate wells in duplicates. Plates were maintained for 24 h in 5% CO_2_ at 37°C. This treatment procedure was used for all experiments in this study.

### 2.3. Measurement of Insulin-Stimulated Glycogen Synthesis

HepG2 cells administered normal (5 mM D-glucose) or high glucose (30 mM D-glucose) were used to determine the effect of GTP on ^14^C-glucose incorporation into glycogen. 1 *μ*Ci ^14^C-glucose solution was added to GTP-treated HepG2 cells for 30 min at 37°C. The reaction was stopped with 30% KOH and transferred into falcon tubes. 30% KOH with 6 mg/mL glycogen was added, and tubes were vortexed carefully and placed on a heating block set at 100°C for 15 min and turned down to 85°C for a further 15 min. 95% ethanol was added to all tubes and vortexed gently until the samples turned uniformly cloudy. Tubes were returned to 85°C heating block for 30 min then into an ice bath. Tubes were left to chill for 15 min to completely precipitate the glycogen. Samples were centrifuged at 2,800 ×g at 4°C for 10 min to pellet glycogen and then ethanol was aspirated, and samples in deionised water were transferred to scintillation vials containing 5 mL scintillation liquid. The samples were counted using a scintillation counter (PerkinElmer Inc, MA, USA). Glycogen synthesis were attained by measuring the rate of incorporation of D-[U-^14^C]glucose into glycogen.

### 2.4. Lipogenesis Assay and Oil Red O Staining

The effect of GTP on lipogenesis in HepG2 cells was tested by a colorimetric assay (Cayman Chemical Company, MI, USA), as previously described [18]. Following standard treatment of HepG2 cells with GTP on coverslips in 24-well plates, SM was removed from the wells with a pipette for staining. 75 *μ*L of Lipid Droplets Assay Fixative (Cayman Chemical Company, MI, USA) was added to each well and incubated for 15 min. Wells were washed with wash solution twice for 5 min each and left to dry completely by placing the plate under a blowing hood. Dye extraction solution was added and wells were gently mixed for 20 min, and the degree of lipogenesis was quantified from lipid droplets in cells by obtaining the absorbance at 490 nm single fixed wavelength with a microplate reader (Bio-Tek Instruments Inc., VT, USA). 

In the separate cell cultures, Oil Red O working solution (Sigma-Aldrich, St. Louis, MO, USA) was added to all wells including the background wells containing no cells and incubated for 20 min. Wells were washed with distilled water several times until the water appeared to be clear (until any pink was not visible). At this point, microscopic images were taken to visualize pink/red oil droplets staining in differentiated cells with the Olympus microscope and an Olympus digital camera (DP70, Tokyo, Japan) using Image-Pro6.2 software (Media Cybernetics, Inc. MD).

### 2.5. Western Blotting

After treatment with GTP-EGCG for 24 h, HepG2 cells were collected and homogenized using RIPA buffer with protease inhibitors (Roche Diagnostics Corporation, IN, USA), and lysates were centrifuged at 14,000 ×g for 20 min at 4°C. Supernatants were collected and protein concentrations were quantified using the Bradford reagent. 

HepG2 lysates were subjected to 7.5% SDS-polyacrylamide gel electrophoresis then transferred to 0.45 *μ*M polyvinyldene difluoride (PVDF) membrane and immunoblotted with primary antibodies phospho-GSK3*β* (Ser9), GSK3*β*, phospho-GS (Ser641), GS, phospho-AMPK*α* (Thr172), AMPK*α*, phospho-ACC (Ser79), ACC (Cell Signaling Technology Inc, MA, USA), and *β*-actin (Santa Cruz, CA, USA) at 1 : 1000 dilution and secondary antibodies (Santa Cruz, CA, USA) at 1 : 10000 dilution. Blots were then developed with enhanced chemiluminescence (ECL) (Pierce, IL, USA) according to manufacturer's instructions. The protein bands were visualized by ChemiDoc XRS systems (Bio-Rad Laboratories, CA, USA) and Quality One 4.6.1 (Biorad) software and density of bands were quantified with the same analysis program. 

### 2.6. Statistical Analysis

Data are presented as the means ± S.E. Comparisons across the variety of treatments were done using one-way ANOVA followed by post-hoc analysis of Tukey's test to determine significant differences between the two treatments using Prism version 4 (GraphPad Software Inc, CA, USA). *P* value < 0.05 was considered statistically significant.

## 3. Results 

### 3.1. GTP-EGCG Increased Glycogen Synthesis in HepG2 Cells

**Figure 2:**
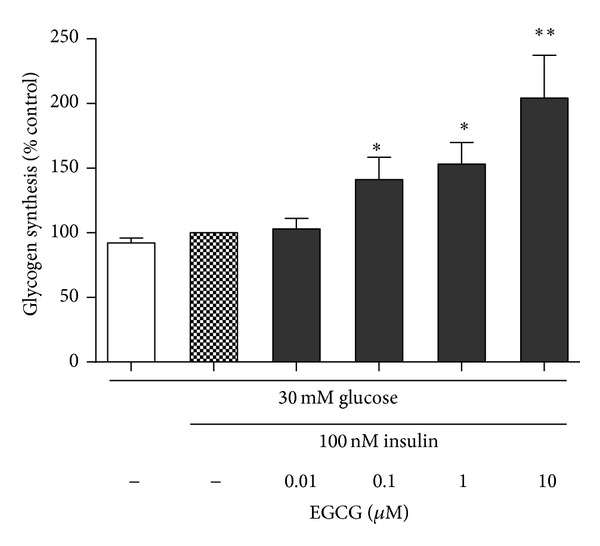
Glycogen synthesis in response to GTP-EGCG treatments in HepG2 cells. The cells were pretreated with high glucose (30 mM) for 24 h then incubated with various concentrations of EGCG (0.1–10 *μ*M) with 100 nM insulin for 24 h. HepG2 cultured with high glucose without insulin was used to confirm that the HepG2 cells became insulin resistant. 1 *μ*Ci ^14^C-glucose solution was added to HepG2 cells for 30 min at 37°C. The results were attained by measuring the rate of incorporation of D-[U-^14^C]glucose into glycogen. Data are means ± S.E. The data are from five separate experiments. *P < 0.05 and **P < 0.01 compared with insulin stimulation alone.

To determine effect of GTP-EGCG on glycogen synthesis, we measured ^14^C-glucose incorporation into glycogen in HepG2 cells pretreated with high glucose (30 mM). Our previous study on cell viability with concentrations of EGCG (0.01–10 *μ*M) showed that EGCG did not exert toxicity in cells (data not shown). Under 100 nM insulin stimulation, glycogen synthesis increased only 2% compared with HepG2 cells cultured with high glucose alone, indicating that high glucose treatment induced insulin resistance in HepG2 cells. Glycogen synthesis was enhanced by 41% and 53% (*P* < 0.05) with 0.1 and 1 *μ*M EGCG. 10 *μ*M of EGCG resulted in a 2fold increase (*P* < 0.01) in HepG2. These data indicate that EGCG increased glycogen synthesis in a dose-dependent manner (Figure 2).

### 3.2. Lipogenesis Was Reduced in GTP-EGCG-Treated HepG2 Cells

**Figure 3:**
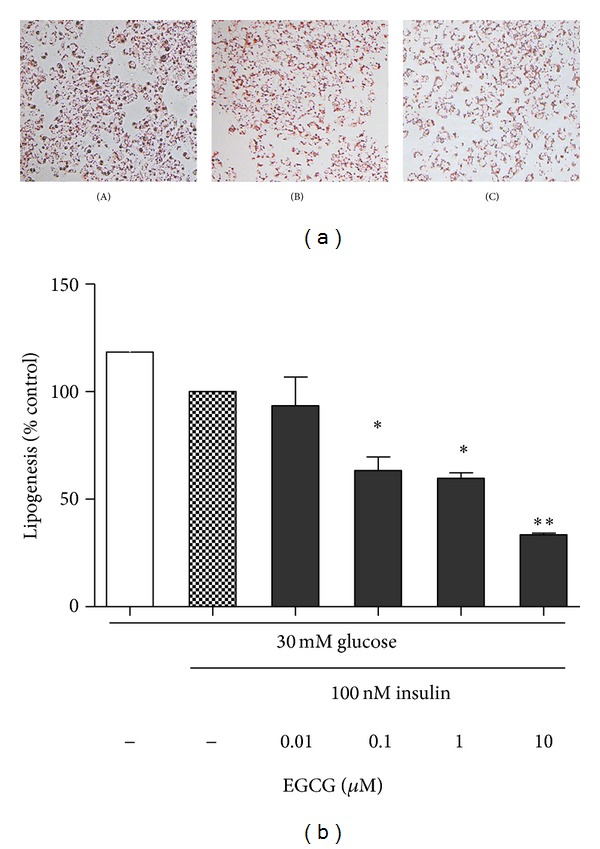
Effects of dose response to GTP-EGCG on lipogenesis in HepG2 cells. HepG2 cells were stained with oil Red O solution, and the dye was extracted from lipid droplets from cells. Images of cells were captured by microscope at 20x original magnification showing lipid accumulation in cells stained by Oil Red O. (A) HepG2 cells cultured in 30 mM glucose, (B) HepG2 cells cultured in 30 mM glucose with 100 nM insulin stimulation, and (C) HepG2 cells cultured in 30 mM glucose with 100 nM insulin stimulation and 10 *μ*M of GTP/EGCG. The degree of lipogenesis was quantified from lipid droplets in cells by measuring the absorbance at 490 nm. The changes of lipogenesis by EGCG treatments were calculated as the percentage of insulin stimulation alone. Data are means ± S.E from five independent experiments. *P < 0.05 and *P < 0.01 compared with insulin treatment alone.

Hepatic lipogenesis is the process by which acetyl-CoA carboxylase (ACC) is converted to fats and involves subprocesses of fatty acid synthesis and subsequent triglyceride (TG) synthesis in the liver. Increased liver fat and elevated hepatic lipogenesis have been demonstrated in obesity and insulin resistance status. To observe effect of GTP-EGCG on lipid deposition, Oil red O staining was used to view lipid droplets in HepG2 cells cultured in high glucose (30 mM) with different treatments.  Figure 3(a) showed a slight decrease in lipid content in the cell culture with 100 nM insulin and a visibly greater reduction of lipid droplets in HepG2 cells treated with 100 nM insulin and 10 *μ*M EGCG. To quantify *de novo* lipid synthesis, HepG2 cells exposed to high glucose were used to determine hepatic lipogenesis with different treatment. At the presence of 100 nM insulin, lipogenesis was reduced by 18% in high glucose treated HepG2 cells but the statistically significant difference was not achieved. GTP-EGCG treatments (0.1, 1 and 10 *μ*M) significantly inhibited lipogenesis in HepG2 cells by 31%, 39% (both *P* < 0.05), and 65% (*P* < 0.01), respectively, compared with HepG2 cells treated with insulin alone (Figure 3(b)). These results indicate that GTP-EGCG improved insulin-medicated lipogenesis in the hepatocytes.

### 3.3. GTP-EGCG Enhanced Hepatic Glycogen Synthesis by Increasing Phosphorylation of Ser9 GSK3*β* and Ser641 GS in HepG2 Cells

**Figure 4:**
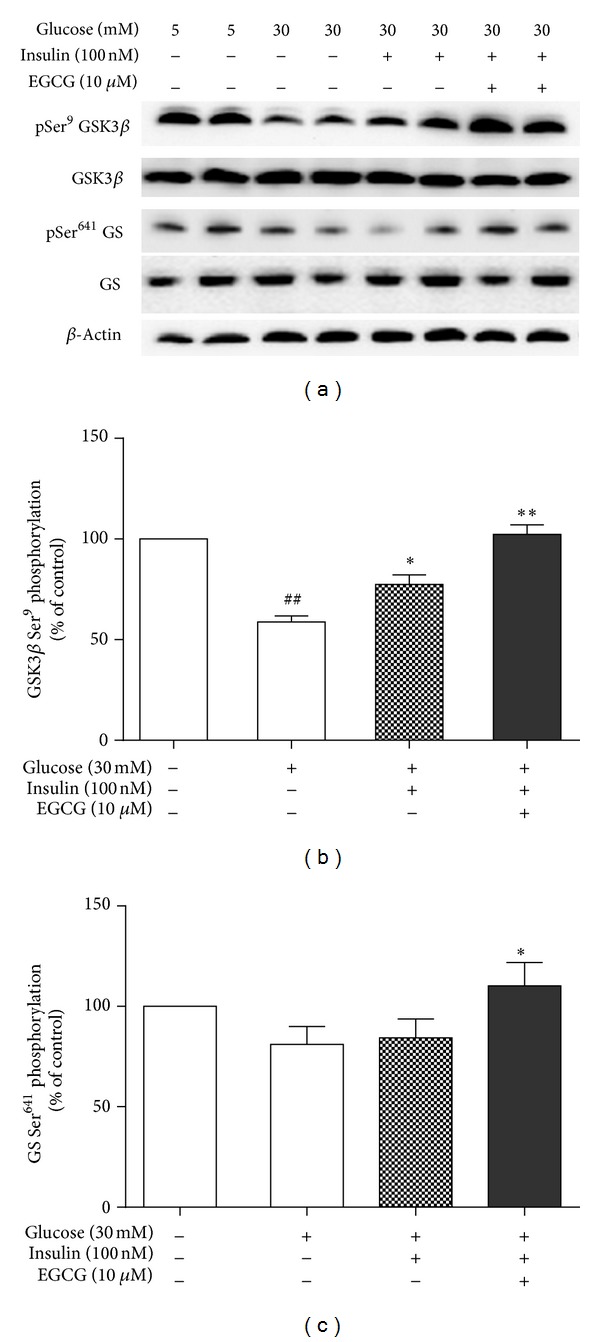
Effects of GTP-EGCG on expressions of phospho-GSK3*β* (Ser9) and phospho-GS (Ser641) in HepG2 cells. The cell lysates were separated on 7.5% SDS-PAGE and incubated with antibodies against phospho-GSK3*β* (Ser9), GSK3*β*, phospho-GS (Ser641), and GS as described. *β*-actin was used as a loading control. A representative western blot of phosphorylation of GSK3*β* and GS from 5 independent experiments is shown. Quantitative data are expressed as mean ± S.E. *P < 0.05 and **P < 0.01 compared with insulin-stimulated HepG2 cells with 30 mM glucose and ^##^ *P* < 0.01 compared with HepG2 cells with 5 mM glucose.

Insulin plays an important role in hepatic glycogen synthesis and in insulin-resistant cellular models; hepatic glycogen synthesis is markedly inhibited [19]. Glycogen synthase kinase 3*β* (GSK3*β*) is a rate-limiting enzyme, which acts as a downstream regulatory switch for numerous signaling pathways such as insulin action, hepatic glycogen synthesis, and lipogenesis. Phosphorylation of GSK3*β* not only activates target enzymes of the insulin-signaling pathway [20] but also regulates hepatic glycogen synthesis by increasing glycogen synthase (GS) expression. Figures 4(a) and 4(b) showed that expression of phospho-GSK3*β* (Ser79) was significantly reduced in high glucose (30 mM) cultured HepG2 cells compared to HepG2 cells with normal glucose (5 mM). Expression of phospho-GS (Ser641) was also impeded by 23% with 30 mM glucose (Figures 4(a) and 4(c)).

Under 100 nM insulin stimulation, reduction of phosphorylation of GSK3*β* in high glucose treated HepG2 cells was improved, and expression of phospho-GSK3*β* (Ser9) was further enhanced by almost 2fold by EGCG (*P* < 0.01) in HepG2 cells with 10 *μ*M EGCG treatment. A significant enhancement of expression of phospho-GS (Ser461) was also observed in EGCG treated HepG2 cells, but insulin alone had no effect on expression of phospho-GS (Ser461) (Figures 4(a) and 4(c)).

### 3.4. GTP-EGCG Inhibited Insulin-Mediated Lipogenesis through Increasing Phosphorylation of Thr172 AMPK*α* and Ser79 ACC in HepG2 Cells

**Figure 5:**
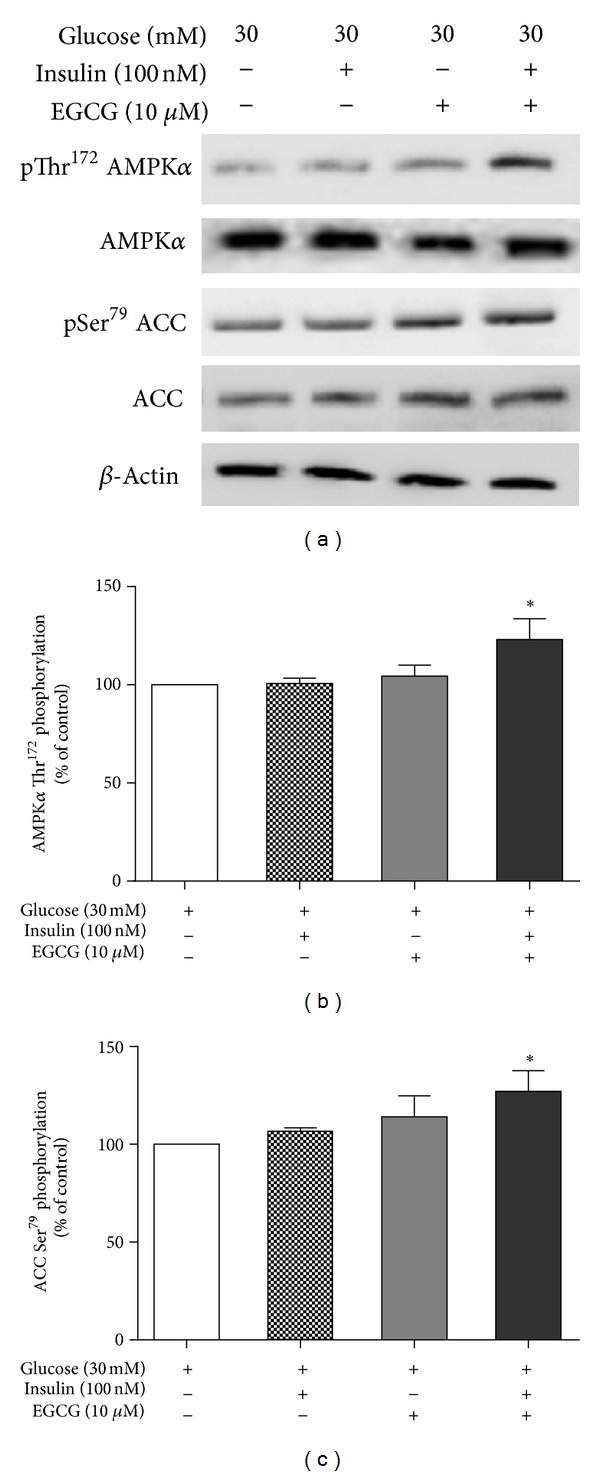
Effects of GTP-EGCG on expressions of phospho-AMPK*α* (Thr172) and phospho-ACC (Ser79) in HepG2 cells. Cell lysates were separated on 7.5% SDS-PAGE and incubated with 1 : 1000 phospho-AMPK*α* (Thr172), AMPK*α*, phospho-ACC (Ser79), and ACC (Cell Signaling Technology Inc, USA) *β*-actin was used as a loading control. A representative western blot analysis from 5 independent experiments is shown (a). Data ((b) and (c)) are expressed as mean ± S.E. (*n* = 5), *P < 0.05, compared with HepG2 cells with 30 mM glucose.

To understand the mechanism of GTP on insulin-mediated hepatic lipogenesis, the effect of 10 *μ*M EGCG on expressions of phosphorylated AMP-activated protein kinase *α* (AMPK*α*) and ACC (two key enzymes involved in hepatic lipogenesis) in HepG2 cells exposed to 30 mM glucose for 24 h was analyzed with western blotting. We found that expressions of phospho-AMPK*α* (Thr172) and phospho-ACC (Ser79) in high-glucose cultured HepG2 cells were decreased when compared with normal glucose cultures (data not shown). Exposure of 100 nM insulin or 10 *μ*M EGCG alone did not change expressions of phospho-AMPK*α* (Thr172) and phospho-ACC (Ser79). Interestingly, expressions of phospho-AMPK*α* (Thr172) and phospho-ACC (Ser79) were significantly increased when HepG2 cells were treated with 100 nM insulin and 10 *μ*M of EGCG (Figure 5), indicating a synergetic effect of EGCG and insulin on phospho-AMPK*α* (Thr172) and phospho-ACC (Ser79) expressions. 

## 4. Discussion

*In vivo* studies have postulated that polyphenolic compounds in green tea reduce body weight, prevent metabolic syndrome, and fatty liver disease through blocking lipid absorption [6–8]. To understand direct effects of GTP and its major active compounds on glucose and lipid metabolism, we determined insulin-mediated glycogen synthesis and *de novo* lipogenesis in HepG2 cells. HepG2 cells were firstly exposed to high glucose to induce insulin resistance, and this was shown by reduced insulin-stimulated glycogen synthesis and elevated lipogenesis. EGCG, the most abundant and potent active compound in GTP [17], was used to calculate a series of mole concentrations to present the total GTP, but the overall results of this study were from total GTP. In this study, we observed dose-response relationship of GTP-EGCG on glycogen synthesis and lipogenesis. 10 *μ*M EGCG under 100 mM insulin-stimulation significantly increased rate of glucose incorporation into glycogen by a 2fold increase in HepG2 cells. Our study also demonstrated that at presence of 10 *μ*M of EGCG, the elevated lipogenesis were normalized in insulin-resistant HepG2 cells. These findings indicate the beneficial effects of GTP-EGCG against metabolic syndrome and diabetes is not only secondary to inhibiting lipid absorption or antioxidant actions [21] but also through its direct action to enhance glycogen synthesis and decrease lipogenesis in insulin-targeted tissues. 

Liver is a major organ involved in insulin-mediated glucose and lipid metabolism. Under insulin-resistant state, hepatic glycogen synthesis is diminished and is also associated with increased lipogenesis, which leads to hyperglycemia and contribute to the development of T2D [22]. In this study, high-glucose cultured HepG2 cells were used to mimic a hepatic insulin-resistant state. The treatments with GTP-EGCG ameliorated the diminished glycogen synthesis indicate that GTP-EGCG is capable of controlling hyperglycemia through reduction of hepatic glucose production. 

To understand the molecular mechanism of GTP-EGCG enhancement of glycogen synthesis, we have detected expressions of phospho-GSK3*β* (Ser9) and phospho-GS (Ser641). GSK3 is a rate-limiting enzyme which acts as a downstream regulatory switch for inactivation of GS leading to reduction of glycogen synthesis [22, 23]. Insulin promotes glycogen synthesis through enhancing expressions of phospho-GSK3*β* (Ser9) and phospho-GS (Ser641). Treatment with GTP/EGCG enhances phospho-GSK3*β*, which mimics insulin's inhibitory effects on GSK3*β*, enhances activity of GS, and subsequently increased glycogen synthesis in HepG2 cells. 

Moreover, GTP-EGCG treatment significantly increased phospho-AMPK*α* (Thr172) and phospho-ACC (Ser79) expressions in HepG2 cells. AMPK and ACC are key enzymes that regulate lipogenesis in the liver [24] and contribute significantly to overall metabolism of lipids. Insulin activates AMPK by promoting its phosphorylation at Thr172 [24] and by direct activation via an allosteric AMP site. Evidence shows that phosphorylation of Thr172, the major stimulatory phosphorylation site of *α* subunit, is essential for AMPK activity [25]. An increase in AMPK*α* phosphorylation in the liver leads to phosphorylation and inactivation of ACC resulting in decreased lipid synthesis, through the biosynthesis of malonyl-CoA from acetyl-CoA, and this may lead to decreased lipid synthesis and regulation of fatty acid oxidation [26].

## 5. Conclusion

We found that GTP-EGCG has direct effects on regulation of glucose and lipid metabolism in high-glucose treated HepG2 cells. Results demonstrate that hepatic glycogen synthesis was significantly upregulated in HepG2 cells with GTP-EGCG treatment through increased phosphorylation of GSK3*β* and GS, which are critical elements in the regulation of hepatic glycogen synthesis *in vivo*. GTP also inhibited hepatic lipogenesis in cells through increased expressions of phospho-AMPK*α* (Thr 172) and phospho-ACC (Ser79). 

In conclusion, our findings showed the beneficial effects of GTP against metabolic syndrome and diabetes through direct enhancement of glycogen synthesis in the liver and decreased hepatic lipogenesis.
